# Identification and impact of discoverers in online social systems

**DOI:** 10.1038/srep34218

**Published:** 2016-09-30

**Authors:** Matúš Medo, Manuel S. Mariani, An Zeng, Yi-Cheng Zhang

**Affiliations:** 1 Department of Physics, University of Fribourg, Chemin du Musée 3, 1700 Fribourg, Switzerland; 2 School of Systems Science, Beijing Normal University, 100875 Beijing, P.R. China

## Abstract

Understanding the behavior of users in online systems is of essential importance for sociology, system design, e-commerce, and beyond. Most existing models assume that individuals in diverse systems, ranging from social networks to e-commerce platforms, tend to what is already popular. We propose a statistical time-aware framework to identify the users who differ from the usual behavior by being repeatedly and persistently among the first to collect the items that later become hugely popular. Since these users effectively discover future hits, we refer them as discoverers. We use the proposed framework to demonstrate that discoverers are present in a wide range of real systems. Once identified, discoverers can be used to predict the future success of new items. We finally introduce a simple network model which reproduces the discovery patterns observed in the real data. Our results open the door to quantitative study of detailed temporal patterns in social systems.

The digital age provides us with unprecedented amounts of information about our society^1^. The collected data are increasingly available at fine temporal resolution which permits us to progress from rudimentary mechanisms in complex systems, such as preferential attachment^2^,^3^,^4^, to their refined versions where the fitness of individual nodes and aging play a fundamental role^5^,^6^. We focus here on data produced by various online systems where users acquire items: buy products, borrow DVDs, or watch videos, for example. This kind of data is at the center of attention of the recommender systems community which aims at predicting items that an individual user might appreciate^7^,^8^,^9^,^10^. The user-item data can be represented and modeled by a growing network where users are connected with the collected items^11^,^12^. Preferential attachment assumes that the rate at which items attract new connections from users is proportional to the number of connections that items already have. Models based on preferential attachment have been applied in a wide range of systems^13^. However, all models to date consider a homogeneous population composed of users driven by item popularity which is modulated by item fitness, aging, or similarity in more elaborate models^5^,^14^,^15^,^16^.

We develop here a statistical framework based on data with time information and use it to show that users in social systems are essentially heterogeneous in their collection patterns. While the majority of users obey preferential attachment and usually collect popular items, some users persistently collect little popular items that later become hugely popular. We introduce a statistical criterion to individuate the users that belong to the latter group—they are referred as discoverers here. We use our framework to demonstrate the presence of discoverers in data from a number of real systems and discuss the relation between discoverers and other related concepts such as opinion leaders^17^,^18^,^19^,^20^,^21^, and innovators^22^,^23^,^24^. Besides none of these concepts providing a full explanation for the behavior of discoverers, the main strength of our contribution lies in a well-defined quantitative method to identify the users that do not follow the omnipresent preferential attachment rule.

We illustrate that identifying the discoverers is of potential use by showing that they can be used to predict the future popular items. Motivated by the fact that the current network growth models cannot reproduce the behavior of discoverers, we generalize a recent model^5^,^25^ by assuming that there are two kinds of users: those who are driven by item popularity and those who are driven by item fitness (fitness of a node is a measure of the node’s attractiveness to a given system^26^). We grow model networks and show that they exhibit similar discovery patterns as those observed in the real data.

## Results

### Identifying discoverers: The statistical procedure

We assume that the input data have a bipartite structure where there are *U* users, *I* items, and *L* links that always connect a user and an item. We label the users with Latin letters (*i*, *j*, …) and the items with Greek letters (*α*, *β*, …) to make the notation transparent. To find the users who act as discoverers of highly popular content, we devise a simple yet effective procedure. We choose a small fraction *f*_*D*_ of the most popular items and track the users who are among the first *N*_*D*_ users connecting with them; here *N*_*D*_ is a small parameter. We label these early links as *discoveries* of the eventually popular content. The number of links created by user *i* and the number of thus-achieved discoveries are denoted by *k*_*i*_ and *d*_*i*_, respectively.

To evaluate whether a user under- or outperforms in making discoveries, we formulate the null hypothesis *H*_0_ that all users are equally likely to make a discovery by each collected item. Denoting the total number of discoveries and links as 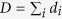 and 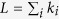, respectively, the probability of discovery for each individual link under *H*_0_ is *p*_*D*_(*H*_0_) = *D*/*L*. Under the null hypothesis, discoveries are independent and equally likely—their number for any given user is thus driven by the simple binomial distribution. This allows us to compute the probability that user *i* makes at least *d*_*i*_ discoveries as


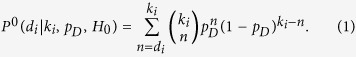


By summing up over *d*_*i*_ discoveries or more, we make sure that the probability *P*^0^ can become very small only if the user makes too many discoveries in comparison with the user’s degree *k*_*i*_ (*d*_*i*_ = 0 results in *P*^0^ = 1). Note that the expected number of discoveries of user *i* is 〈*d*_*i*_〉 = *p*_*D*_*k*_*i*_ and the total expected number of discoveries is therefore


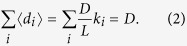


The binomial distribution for the number of discoveries by individual users and the real number of discoveries are thus compatible with each other. Note that the null hypothesis effectively decouples the users whose discoveries are assumed to be independent of the discoveries made by the others. While this is not strictly true on a link-by-link basis—a user sometimes creates a link at a moment when there are no discoveries possible—it still holds for each user overall because every user makes several links and, moreover, users are free to choose the time when they create links.

To quantify the extent to which is the behavior of user *i* incompatible with the null hypothesis, we introduce user *surprisal*^27^ (also referred to as self-information^28^)


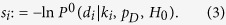


A high surprisal value indicates that the user’s success is unlikely under *H*_0_ (in principle, *s*_*i*_ is the logarithm of the hypothesis *p*-value computed for an individual user). The lowest possible surprisal value *S*_*i*_ = 0 and the highest possible surprisal value *S*_*i*_ = −*k*_*i*_ ln *p*_*D*_ are achieved when *D*_*i*_ = 0 and *D*_*i*_ = *k*_*i*_, respectively. To evaluate whether a user’s discovery behavior is compatible with the null hypothesis, we compare it with the average largest surprisal value in bootstrap realizations of the system^29^ (see Materials and Methods for details). Any user whose real surprisal is higher than this value is referred to as *discoverer*; the number of discoverers is labeled as *U*_*D*_.

### Discoverers in real data

**Figure 1:**
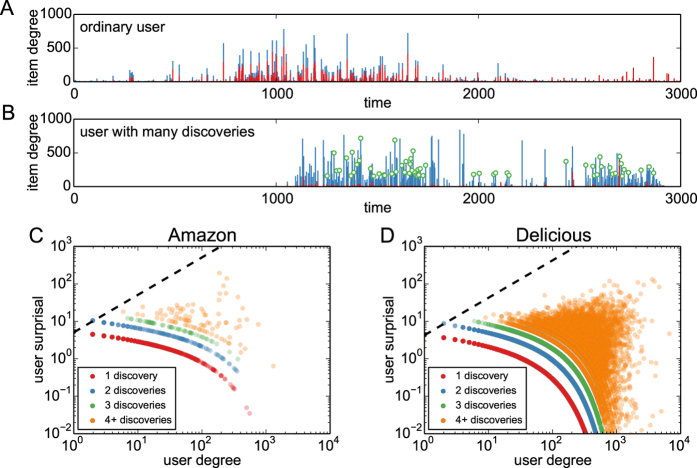
Discoveries and user surprisal in real data. (**A,B**) A comparison of the linking patterns of an ordinary user and a “discoverer” in the Amazon data. Each bar here corresponds to a collected object where the red and blue part show item popularity at the time when the user collected it and the final popularity, respectively. Green circles mark those collected items that are eventually identified as discoveries (see the definition in text). (**C,D**) Scatter plots of user degree and user surprisal in the Amazon and Delicious data. Users are color-coded according to their number of discoveries. The dashed lines mark the maximal achievable user surprisal at a given user degree. All results are for *f*_*D*_ = 1% and *N*_*D*_ = 5.

Figure 1 shows the discovery patterns and user surprisal in the datasets on DVD purchases at Amazon.com and personal bookmark collections at Delicious.com (see Materials and Methods for description of the datasets). Panels 1A and 1B compare the linking patterns of two Amazon users of different surprisal. The “ordinary user” either collects popular items late or collects unpopular items and thus achieves no discoveries. By contrast, the “user with many discoveries”, though only active later during the dataset’s timespan, is frequently among the first to collect eventually popular items and achieves 59 discoveries in 283 links whereas the overall discovery probability is *p*_*D*_ ≈ 0.5% which for the given number of links corresponds to 1.4 discoveries on average. Panels 1C and 1D further show the degree and surprisal values in the analyzed data. While the maximal possible surprisal value of an individual user grows linearly with user degree (depicted with dashed lines), user activity alone is no guarantee of high surprisal and top surprisal values are achieved by some moderately active users. One can see here that when the number of discoveries is fixed, the surprisal value decreases with user degree. Note that the highest observed surprisal values correspond to particularly low *P*^0^ probabilities of 10^−131^ and 10^−56^ for the Amazon and Delicious data, respectively.

**Figure 2:**
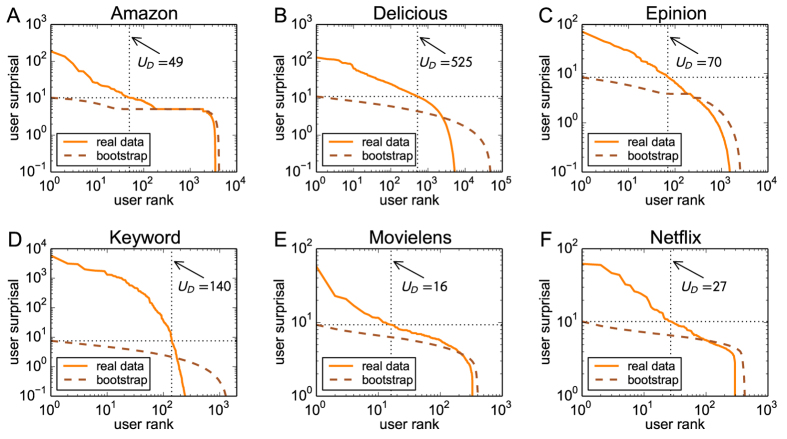
User surprisal in real data and in bootstrap. All results are for *f*_*D*_ = 1% and *N*_*D*_ = 5.

Results of the bootstrap analysis in Fig. 2 show that the largest surprisal values in bootstrap realizations sampled under *H*_0_ are never as high as the largest surprisal in all six analyzed datasets. For *f*_*D*_ = 1% and *N*_*D*_ = 5, there are 49 and 525 identified discoverers in the Amazon and Delicious data, respectively (0.01% and 0.49% of all users, respectively). Figures in Supplementary Materials (SM) further demonstrate that there is no particular time bias in the discovery patterns (e.g., discoverers are not those who happen to be active earlier or longer than the others) and the discoveries are made continuously during the system’s lifetime (Figures S1 and S2, respectively). In other words, discoverers are persistent in their behavior. While numerical values of surprisal depend on parameters *f*_*D*_ and *N*_*D*_, the resulting ranking of users by their surprisal is rather stable (see Figure S3). Figure S4 finally demonstrates that the ranking of users by their surprisal does not change considerably when part of the data is taken into account. We can conclude that the null hypothesis of user homogeneity needs to be rejected because some users are indeed significantly more successful than the others in early collecting eventually popular items. This phenomenon is not restricted to particular conditions and consistently emerges in systems where individuals are free to choose from many heterogeneous items.

### Using user surprisal to choose the future popular items

**Figure 3:**
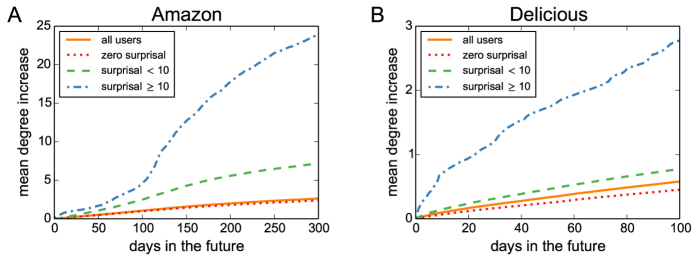
Future degree evolution of the target items collected by users of different surprisal in Amazon (**A**) and Delicious (**B**) data. The items collected by high surprisal users become significantly more popular (according to the Mann-Whitney test) than target items collected by users of low or zero surprisal. The popularity ratio between the high and zero surprisal group at the end of the future time window is 10.0 and 6.1 for the Amazon and Delicious data, respectively.

We next investigate whether the presence of users who make discoveries more often than the others is of some practical significance. To this end, we generate multiple data subsets and in each of them define young items with exactly one link as the target items whose future popularity is to be predicted (see SI for details). Since the information on these items is extremely limited (only one link from one user is attached with them) and the social network of users as well as item metadata are either absent in the studied systems or not known to us, traditional methods for prediction of popularity of online content cannot be used here^30^,^31^,^32^. We divide users in each subset into three groups: zero, low, and high surprisal users (the threshold between low and high surprisal is set to 10 which is close to the average highest surprisal value in bootstrap in both data sets). The data that come after a given subset are then used to evaluate the future degree evolution for the target items collected by users from different groups. Figure 3 shows that the target items chosen by users of high surprisal become significantly more popular than those chosen by users of zero or low surprisal. This demonstrates that surprisal not only quantifies users’ past behavior but, thanks to the persistence of the discovery behavior, it also has predictive power.

### What makes a discoverer

Are there some user features that directly contribute to the user appearing as a discoverer in the above-described analysis? As shown in Fig. 1 (panels C and D), user degree is little correlated with surprisal. Collecting the items that eventually become popular also does not contribute to user surprisal in the Amazon dataset: no discoverer is found among the 1000 users with the highest average final degree of the collected items. The same is true for collecting little popular items in the Amazon dataset: among the 1000 users with the lowest average current degree of the collected items at the time when they have collected them, there are again no discoverers. In the Amazon data, we have the information on the number of users who find a review useful, which allows us to study the possible correlation between the average level of usefulness of a user’s reviews and the user’s surprisal value. However, we find no significant correlation which suggests that well-written and informative reviews do not contribute to the success of discoverers.

Another possible explanation lies in the discoverers being more influential than other users (see refs 19, 20, 21 for recent research of influential users) and the items they collect are thus likely to become popular. However, most of the systems that we analyze here lack any explicit mechanism for users to exert influence over the others, especially on such short time scales as we speak of here (we use *N*_*D*_ = 5 through the paper, which means that only the first five users are awarded a discovery for collecting a relevant item). This agrees with the finding that easily influenced individuals contribute to the rise of exceptionally popular items more than so-called influentials^19^. The situation is different in the Amazon dataset because of the Amazon’s Vine program which gives a small number of users advance access to not-yet-released products—those users are then in an obviously privileged position to write an early review and be awarded a discovery. Nevertheless, less than 30% of the identified discoverers are marked as members of the program and, conversely, many program members are not identified as discoverers.

To study the issue of potential user influence more closely, we turn to the dataset from the Yelp academic challenge, round 4 (see http://www.yelp.com/academic-dataset for more information). The advantage of this dataset is that unlike the datasets analyzed here, Yelp data feature both the bipartite user-item network as well as the social user-user network (the Delicious web site also allowed the users to form friendship links but unfortunately we do not possess the social network data). The input data contains 252,898 users, 42,153 items (which in this case represent businesses), 955,999 friendship links, and 1,125,458 reviews in the integer scale from 1 to 5; the time stamps run from 0 to 3558 (measured in days). We only keep the users who have at least one friend and one authored at least one review. As for the other datasets, we use the rating threshold of four to obtain an unweighted bipartite network and focus on a subset of the data (in this case the the evaluations from days 1000 until 3499; we thus ignore the rather long initial period of 1000 days which aims at avoiding the notorious items that existed before day 0 and awarding discoveries for them would therefore be unjust). We finally have a dataset with 80,840 users, 33,661 items, 348,060 user-item links and 674,231 directed user-user links.

As in the other reported datasets, also the Yelp data features discoverers: the largest user surprisal value is 21, the average highest surprisal in bootstrap realizations is 9.7, and the number of identified discoverers is 30. The discoverers have on average 7 friends which is less than the average number of friends in the whole Yelp dataset (10.3). We can conclude that in the Yelp data, users with many social contacts are in no way more successful in achieving discoveries than users with few social contacts. Social influence thus cannot be the sole explanation for the observed discovery patterns. It is also worth to note that among the 30 identified discoverers, there is not a single explicit social link in the Yelp data, which shows that the discoverers are no privileged closed group. Other network centrality metrics, such as betweenness and *k*-shell^12^, show similar negative results.

In summary, the discoverers identified by the proposed statistical method do not appear to share any particular trait except for the defining one: they are disproportionately often among the first ones to collect the items that eventually become very popular. Note that this is similar to the existing notion of innovators who are among the first ones in the adoption curve of products or innovations^22^. The crucial difference lies in considering the temporal dimension and the persistence of user behavior—discoverers are at the start of the adoption curve for many successful items. By contrast to the standard theory of innovations by Rogers^22^ where innovators have the highest social status, are social, and interact with other innovators, we found that none of these characteristics apply to the identified discoverers.

### Network model

Since none of the existing network growth models based on preferential attachment is able to reproduce the observed discovery patterns, we propose a simple model that fares better in this respect. In the model, we assume that some items are inherently more fit for a given system than the others and thus have higher chance of becoming very popular in the long run. Network models with node fitness have been studied in the past^15^,^26^,^33^ and they have been used to model various systems such as the World Wide Web^34^, citations of scientific papers^5^,^35^, and an online scientific forum^25^, for example. Unlike the existing models, we then assume that the users differ in how they perceive item fitness and choose the items for their collections. While the first group of users are driven by item popularity and thus mostly ignore new and little popular items, the second group of users are driven by item fitness. Discoverers then emerge among the users in the latter group because: (1) fitness-driven users are consistently among the first ones to collect items of high fitness, (2) high fitness items are likely to become very popular, (3) active fitness-sensitive users have the potential to achieve many discoveries and eventually be identified as discoverers by the statistical procedure that we propose here.

We generate artificial bipartite networks with *U* users where the number of items gradually grows from a small number *I*_0_ to *I* (we use *U* = 4000, *I*_0_ = 50, and *I* = 8000 here). There are *U*_*F*_ fitness-sensitive users and the remaining *U* − *U*_*F*_ users are popularity-driven. Each user is further endowed with a level of activity which determines the rate at which users collect new items. While one can vary the distribution of activity among the users to model a broad range of real systems, user activity values are for simplicity drawn from the uniform distribution [0, 1] here. Item fitness quantifies how suitable and attractive is an item to the given system and its users; fitness values *f*_*α*_ are drawn from the power-law distribution with the lower bound *f*_min_ = 1 and exponent 3. As the analytic computation in SM shows, a power-law fitness distribution directly translates into a power-law distribution of item popularity (see ref. 36 for a similar direct relation between an input power law and an output power law in network modeling). Our choice of the item fitness distribution thus allows us to mimic real systems where the distribution of node popularity (degree) is often broad, typically power-law or log-normal^37^. Time at which item *α* has been added in the system is denoted as *τ*_*α*_. New links are added regularly until the final network density *η* is achieved; the total number of links is thus *L* = *ηUI*. To reach *I* items before all links have been added in the network, new items are added every *L*/(*I* − *I*_0_ + 1) steps.

In the simulation, one user-item link is added in each time step. The user who creates this link is chosen from the pool of users with probability proportional to user activity. If a fitness-driven user *i* creates a link at time *t*, the probability of choosing item *α* is proportional to


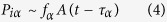


where *A*(*t* − *τ*_*α*_) = exp[−(*t* − *τ*_*α*_)/*θ*] is an aging factor (see refs 5 and 25 for the original model of network growth with heterogeneous fitness and aging). Consequently, *θ* is a typical lifetime at which item attractiveness decays; we use *θ* = 1000 steps which is neither too quick (in which case the high-fitness items do not have sufficient time to attract many links and the resulting degree distribution is thus very homogeneous) nor too slow (in which case a strong bias towards old items develops and the fitness-popularity correlation is low). If a popularity-driven user *i* creates a link at time *t*, the probability of choosing item *α* is proportional to


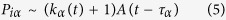


where *k*_*α*_(*t*) is the degree (popularity) of item *α* at time *t*. The additive term in *k*_*α*_ + 1 is necessary to allow items of zero degree (every item is introduced in the system with zero degree) to gain their first links. Multiple links between a given user and an item are not allowed.

Note that two consumer groups—innovators and imitators—are assumed also by the Bass model^18^ which constitutes a seminal model for the diffusion of innovations. However, the original Bass model does not consider competition among the items and the link between an item’s final popularity and its properties. Because of its focus on individual items, the Bass model cannot reproduce persistent discovery patterns and thus we do not use it here to model the discovery patterns found in real data.

### Results on model data

**Figure 4:**
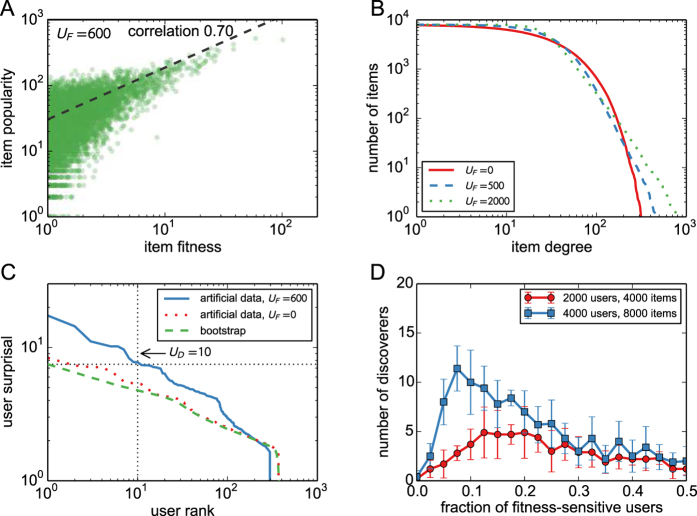
User surprisal and discoverers in artificial model networks. (**A**) The relation between item fitness and popularity as well as the cumulative distribution of item popularity (*U*_*F*_ = 600). (**B**) Item degree distributions for various values of *U*_*F*_. (**C**) The plot of user surprisal in artificial data (*U*_*F*_ = 0, 600) and in bootstrap (this curve is independent of *U*_*F*_). (**D**) The dependence of the number of discoverers identified by the proposed statistical procedure on *U*_*F*_/*U*. For comparison, we also show here results for 2,000 users and 4,000 items.

Simulation results for the artificial model are presented in Fig. 4. Panel A shows that when a significant number of users are sensitive to item fitness (here *U*_*F*_ = 600), there is considerable correlation between between item fitness and popularity in the resulting networks. As *U*_*F*_ decreases, this correlation gradually vanishes because we assume that the popularity-sensitive users ignore item fitness. As shown in panel B, the distribution of item popularity is indeed rather broad and displays a power-law tail when *U*_*F*_ is positive which agrees with the approximate analytical solution in SM. Panel C demonstrates that when *U*_*F*_ is positive, user surprisal computed in model data differs from the bootstrap surprisal profile in the same way as we have shown in Fig. 2 for the real data. The number of identified discoverers as a function of the number of fitness-sensitive users is displayed in panel D. The dependence is notably non-monotonous. When *U*_*F*_ is small, the correlation between item fitness and popularity is low and many of the popular items that are used to assign discoveries are thus of low fitness; the fitness-sensitive users thus fail to achieve many discoveries and the resulting *U*_*D*_ is close to zero. As *U*_*F*_ grows, the fitness-popularity increases and so does *U*_*D*_ but eventually, there number of fitness-sensitive users is too large for the number of available discoveries and *U*_*D*_ declines. For intermediate values of *U*_*F*_, the numbers of identified discoverers are significant and we can thus conclude that the proposed simple model is able to reproduce the discovery patterns observed in real data. The observed fraction *U*_*D*_/*U* which gets as high as 0.03% at *U*_*F*_ = 300 is similar to that found in the Amazon data.

Note that the groups of fitness-driven users and discoverers are in general not the same. While in the current setting, all discoverers identified using the proposed statistical framework are fitness-driven, only a small fraction of fitness-driven users are identified as discoverers (in Fig. 4D, for example, *U*_*F*_ = 600, yet 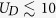). There are various reasons why a fitness-driven user does not become a discoverer: the user is not active enough, or by chance becomes active at moments when there are no relevant items (that is, little popular high-fitness items) available and hence no discoveries can be made, or simply fails to connect with the available relevant items because of the probabilistic network growth mechanism. The fact that discoverers are found in the model data is thus not automatic and the number of statistically significant discoverers depends strongly on model parameters.

It has been demonstrated that in real systems, the popularity of items is path-dependent and sensitive to system design and possible external factors^38^,^39^ which questions the choice of the most popular items as the items for which discoveries are awarded to the users. The analysis of model data allows us to return to this important point equipped with better understanding of both the statistical procedure and the systems on which it is applied. Despite the fact that the correlation between item fitness and popularity is far from perfect (see Fig. 4A), we find discoverers in the model data and it can be verified that almost all of them are indeed fitness-sensitive users. This high robustness of the model to a sub-optimal choice of relevant items is due to the fact that when some popular items are actually of low-fitness, fitness-sensitive users simply ignore them. By contrast, the popularity-sensitive users gain some discoveries for these inferior popular items but since these users are typically in majority by a wide margin, the limited number of discoveries that distributes among them is not sufficient to yield significant surprisal values. An imperfect choice of the relevant items thus reduces the useful signal for discoverer identification, yet it creates only a weak false signal for popularity-sensitive users.

## Discussion

In this article, we introduce discoverers as the users in data from real systems who significantly outperform the others in the rate of making discoveries, *i.e.* in being among the first ones to collect items that eventually become very popular. We develop a statistical framework to identify the discoverers and use it to demonstrate that they can be found across a number of online systems where users have the freedom to choose from a large number of possible items. The proposed approach is suitable to any data with time information. Evidence for discovery behavior in monopartite networks (work in progress) shows that our approach is applicable and relevant to an even broader range of systems than those studied here. The ability to identify the discoverers is shown beneficial for predicting the future popularity of items as well as for ranking the users.

We stress again that the classical concepts of social leaders or innovators who have high social status or are well positioned in the social network, extensively studied in the past^17^,^18^,^21^,^22^, do not provide an explanation for the presence of discoverers who do not share any advantageous or privileged social position and achieve discoveries consistently over time. Our work demonstrates the presence of discoverers in social systems and at the same time calls for a deeper understanding of their behavior and roles. To quantify the level to which a user’s discovery performance is due to some external influence (such as the above-mentioned Amazon Vice program) is just one of the steps towards understanding the phenomenon of discoverers.

Motivated by the generality of the observed phenomenon and a lack of direct ways for an individual to influence other users in the systems studied here, we search for a general mechanism to model the discovery behavior. To this end, we generalize the preferential-attachment network growth model with fitness and aging^5^ by assuming that not only the item nodes differ in their fitness but also the user nodes differ in their sensitivity to item fitness. In the model data, fitness-sensitive users recognize the high fitness items, collect them, and these items then often eventually become very popular due to their high fitness. While the model reproduces the discovery patterns found in the real data, we emphasize that the main goal of the model is to show that the reported discovery patterns can be modeled based on a small variation of the existing network growth models. A comprehensive quantitative and qualitative analysis of various possible reasons for the presence of discoverers in real data as well as a study of model formulations that best agree with real data remain as future research challenges.

## Materials and Methods

### The bootstrap analysis

To evaluate whether a user’s discovery behavior is compatible with the null hypothesis, we use parametric bootstrap^29^. Using the discovery probability *p*_*D*_, we generate the number of discoveries under *H*_0_ for each user according to Eq. (1), compute the corresponding bootstrap surprisal value, and consequently compute the largest bootstrap surprisal value found for any of the users. By repeating this procedure many times, we find the average largest surprisal value in bootstrap 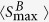 (we use 10,000 independent bootstrap realizations to compute this average; the more realizations, the more precise the estimate). Any user whose real surprisal is higher than this value is referred to as *discoverer*; the number of discoverers is labeled as *U*_*D*_. Bootstrap surprisal values are further used for comparison with real user surprisal values in Fig. 2.

### Data description

To properly detect discoverers in a given dataset, we need to avoid the items that have actually appeared before the dataset’s start. The reason for this is that if such an item is selected as the target item, we award users who collect it first with discoveries despite the fact that the item has existed before. To prevent that, we always select a subset of the given dataset by specifying its start and end point, and discard the items that have appeared before the subset’s start point. When the start point is sufficiently “late”, old items effectively do not appear in the subset. Since the observed discovery patterns are robust with respect to the data (see SM, Figure S4), the precise choice of the start and end point does not alter the results qualitatively. Properties of the used subsets are summarized in Table 1.

Amazon.com is a leading online retailer. We obtained the Amazon DVD review data from snap.stanford.edu/data/web-Movies.html^40^. After data cleaning (merging distinct items which actually correspond to the same product—different releases of a DVD are the typical example of this phenomenon—and removing duplicate reviews), there are 1,901,110 reviews in the integer scale 1–5 from 889,066 users for 141,039 items. While the data span 5,546 days (August 1997-October 2012), we only use the data from days 2,000 to 5,000 because the rest of the data shows comparably low activity of users. To obtain an unweighted bipartite network, we neglect all reviews with rating 3 or less and represent all reviews with rating 4 or 5 as links between the corresponding user and item. After this operation, there are 713,581 links whereas 406,275 users and 76,205 items have at least one link.

Delicious.com is a web site that allows users to store, share, and discover web bookmarks. Delicious bookmark collections were obtained by downloading publicly-available data from the social bookmarking website delicious.com in May 2008. Due to processing speed constraints, we randomly sampled 50% of all users available in the source data and included all their bookmarks. To avoid the possible ambiguity of various web addresses pointing to the same web page, reduce the number of items and thus increase the data density, bookmarks are represented only by their base www-address without the initial protocol specification, possible leading “www.” and the trailing slash (*e.g.*, http://www.edition.cnn.com/US/ is modified to edition.cnn.com); each www-address is then represented as an item-node and connected with the users who have collected it. Time stamps are counted in hours from 01/09/2003 and run from 0 to 36,027. For the same user activity reasons as in Amazon, we only use the data from hours 15,000 to 35,000. There are 107,810 users, 2,435,912 items and 9,322,949 links in the resulting data. We have analyzed also data where the full address hierarchy is preserved (*e.g.*, edition.cnn.com/US instead of the previously mentioned edition.cnn.com) and found the same behavior as presented here.

Epinions.com is a consumer review web site. We obtained the Epinion data from konect.uni-koblenz.de/networks/. The original data comprise 120,492 users, 755,760 items and 13,668,320 ratings. Time span of the data is from 9 January 2001 to 29 May 2002. In the raw data, the time stamps exhibit a periodic pattern with respect to link order. In addition, many links appear at the starting day of the data. To avoid these two problems, we use only links ranked from 12,276,827 to 13,213,749 in the original data. Since ratings are in the integer scale from 1 to 5, we apply the same threshold mechanism as in the Amazon data. The final subset contains 17,542 users, 32,482 items and 753,392 links. Time span of the subset is from 16 January 2001 to 29 May 2002 (499 days in total).

Taobao.com is the biggest Chinese e-commerce platform. The produce keyword data were crawled from the web site via its open API. On Taobao.com, vendors can use keywords to describe their products and well-chosen keywords can contribute to their products being ranked at the top of customers’ search results. Vendors have to pay a price for using keywords and the price of a keyword depends on the keyword’s popularity—vendors thus have an incentive to invent new keywords or early adopt already existing keywords. The data comprise 2,824,853 links between 1,523 online retailers and 915,271 keywords that they attached to their products. Time span of the data is from 12 November 2009 to 21 June 2014 (40,360 hours in total).

Movielens.org is a non-commercial web site with personalized movie recommendations. We obtained the Movielens data from grouplens.org/datasets/movielens/. The original data comprise 10,000,054 ratings from 71,567 users to 10,681 movies in the online movie recommender service MovieLens. Since ratings are in the integer scale from 1 to 5, we apply the same threshold mechanism as in the Amazon data. Time span of the data is from January 1995 to January 2009 (122,634 hours in total). We use the subset from hour 40,000 until the end of the data. The final subset contains 2,132,128 links between 44,548 users and 7,974 items.

Netflix is an international DVD rental and on-demand media streaming provider. Netflix DVD rating data were made available for the Netflixprize contest and can be still downloaded from www.netflixprize.com/. The original data comprise 100,481,826 ratings from 480,189 users to 17,770 movies in the online DVD rental website Netflix. Since ratings are in the integer scale from 1 to 5, we apply the same threshold mechanism as in the Amazon data. Time span of the data is from January 2000 to January 2006 (2242 days in total). We use the subset from day 500 to day 1,500 to constraint the data size. The final subset contains 2,775,772 links between 115,131 users and 7,351 items.

### Evaluation of future degree evolution

We choose subsets of time span *T*_*S*_ by choosing their starting time *T*_*X*_ at random from the range [0, *T*_*W*_ − *T*_*S*_ − *T*_*F*_) where *T*_*W*_ is the time span of the whole dataset and *T*_*F*_ is the length of the future time window, over which we observe the future degree increase of items. A given subset is then used to compute surprisal of all its users. We further choose all items that have received exactly one link and they have appeared at most *τ*_max_ before the subset’s end time (this represents young and yet unpopular items) as items of interest. We then track all links that are attached to the items of interest in the future time window of length *T*_*F*_ (i.e., these links are not part of the subset which was used to compute user surprisal values). This allows us to compute the average degree of these items as a function of time. Results are further averaged over 100 subsets defined by their *T*_*X*_ value.

We use the subset parameter values *T*_*S*_ = 2,000 days, *T*_*F*_ = 300 days, and *τ*_max_ = 20 days for the Amazon data and *T*_*S*_ = 300 days, *T*_*F*_ = 100 days, and *τ*_max_ = 2 days for the Delicious data, which accounts for different dynamics of these two systems. While the chosen values influence the detailed shape and relative heights of the curves reported in Fig. 3, the main result that the items collected by high surprisal users become on average more popular than the items collected by users of zero or low surprisal holds always.

## Additional Information

**How to cite this article**: Medo, M. *et al.* Identification and impact of discoverers in online social systems. *Sci. Rep.* **6**, 34218; doi: 10.1038/srep34218 (2016).

## Supplementary Material

Supplementary Information

## Figures and Tables

**Table 1:** Basic properties of the studied datasets.

Dataset	Users	Items	Links	Time span	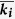	*K*_*i*_	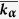	*K*_*α*_	*k*_*D*_
Amazon	406,275	76,205	713,581	3,000 days	1.8	1,296	9.4	790	127
Delicious	107,810	2,435,912	9,322,949	20,000 hours	86.5	6,582	3.8	7,014	40
Epinions	17,542	32,482	753,392	499 days	42.9	5,809	23.2	508	154
Keyword	1,523	915,271	2,824,853	40,360 hours	1855	23,775	3.1	227	32
Movielens	44,548	7,974	2,132,128	82,624 hours	47.9	2,419	267	18,858	3,852
Netflix	115,131	7,351	2,775,772	1,000 days	24.1	959	378	26,700	8,256

The time span column specifies both duration and time resolution of the datasets. 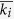 and 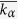 are the mean user and item degree, respectively. *K*_*i*_ and *K*_*α*_ are the largest user and item degree, respectively. *k*_*D*_ is the smallest degree upon which an items is considered as one of items that are to be discovered when *f*_*D*_ = 1%.
