# Supplementary material for: Identification and impact of discoverers in online social systems
Source: Sci Rep. 2016 Sep 30;6:34218. doi: 10.1038/srep34218 (PMC5043231; doi:10.1038/srep34218)
Supplement: Supplementary Information [file srep34218-s1.pdf]

# Identification and impact of discoverers in online social systems

## Supplementary Materials

M. Medo, M. S. Mariani, A. Zeng, Y.-C. Zhang

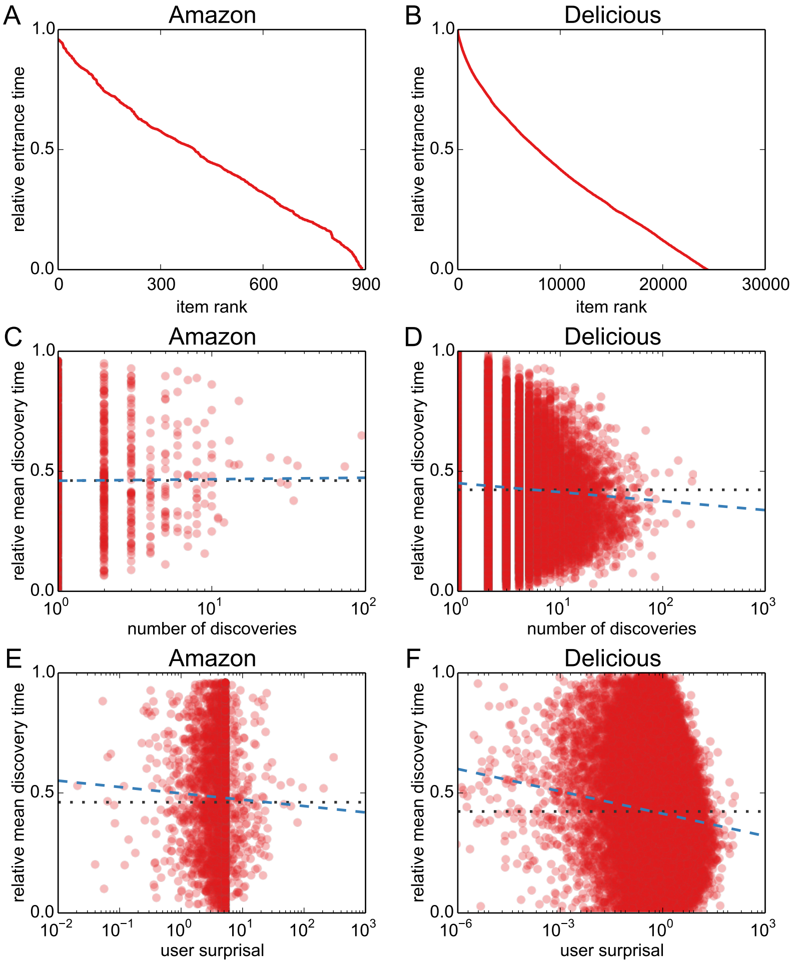


Figure S1: **Temporal distribution of discoveries.** We investigate here whether discoveries and surprisal are not strongly biased towards, for example, the early period of the analyzed data when the number of items was small and thus it was maybe easier to make discoveries. To this end, we show the Zipf plots of the relative entrance time of target items (the relative entrance times of 0 and 1 correspond to the beginning and end of the data set). Nearly straight lines in panels A and B indicate that target items are distributed rather uniformly through the data time span. Panels C and D show the mean discovery time (again in relative units) of individual users plotted the number of discoveries achieved by them. The dotted line represents the mean discovery time averaged over all users. The dashed line represents a linear fit of the data for individual users in the log-linear plane. Panels E and F show the mean discovery time of individual users against their surprisal. See Figure S2 for more detailed information about the discovery patterns of top 20 users in both number of discoveries and surprisal.

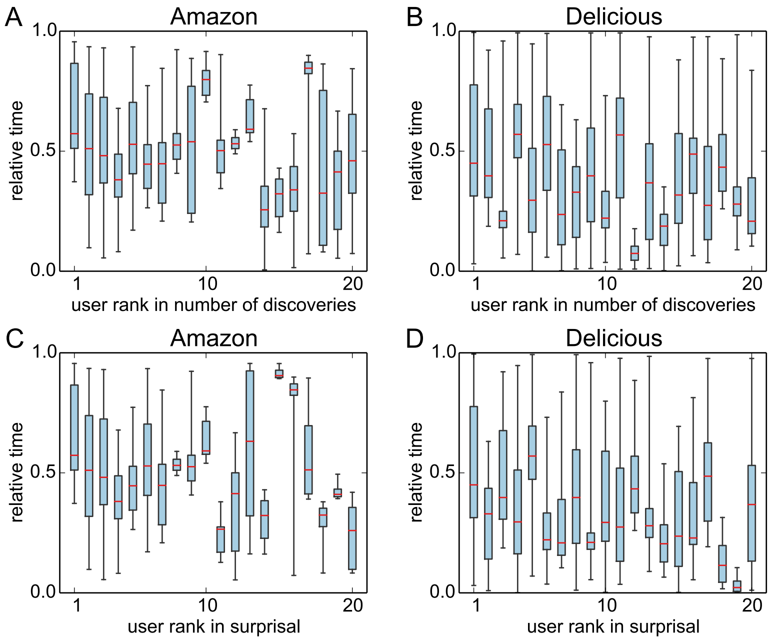


Figure S2: **Temporal distribution of discoveries for top 20 users.** To uncover possible time bias in the discovery patterns of users, we show here box plots for discovery times by individual users who are ranked among the top 20 users either in number of discoveries (A, B) or in surprisal (C, D). The boxes represent the first and third quartile of the discovery times for each individual user; the bands represent median values; the whiskers represent the minimum and maximum values. One can see here that discoveries are spread over a substantial time period for majority of top users with only a few users achieving a substantial fraction of their discoveries at the very beginning of the data (the only exceptions are user #19 in surprisal and user #12 in number of discoveries, both in the Delicious data). We can conclude that high numbers of discoveries and surprisal achieved by some users are not due to their privileged position.

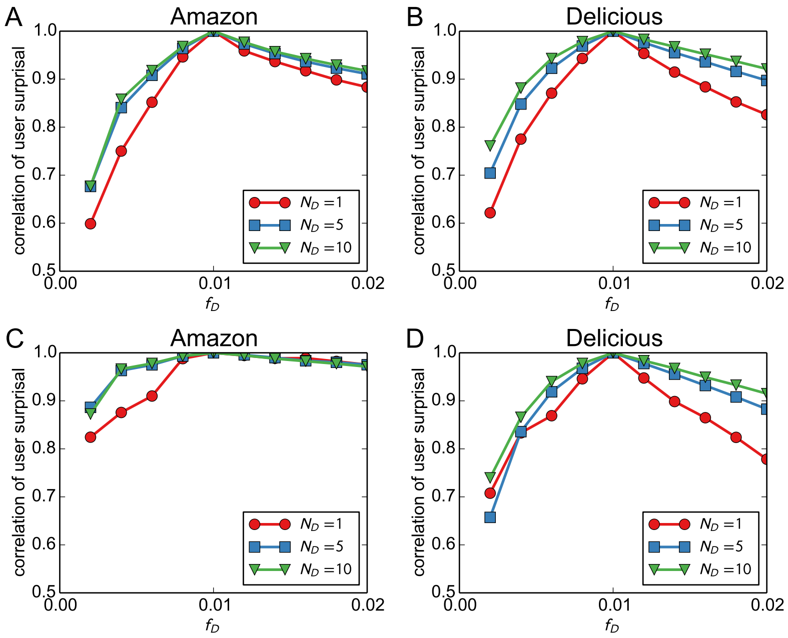


Figure S3: **Stability of user surprisal values with respect to parameters.** In the main text, we choose $f_D^* = 1\%$ and $N_D = 5$ for both Amazon and Delicious data. To investigate the effect of these two parameters on user surprisal values, we first compute the vector of user surprisal values for $f_D^*$ and denote it $\boldsymbol{S}^*$. We then calculate the vector of surprisal values $\boldsymbol{S}$ for any different $f_D$ and compute the Pearson correlation coefficient $r(\boldsymbol{S}^*, \boldsymbol{S})$ which is then shown in panels A and B. The procedure is the same in panels C and D, except for the computation of Pearson correlation coefficient only over users whose surprisal in $\boldsymbol{S}^*$ is greater than 10 (we focus in this way on users who matter most from the perspective of their discovery ability). Results for various values of $N_D$ (recall that $N_D$ first links attached to a target item are marked as discoveries) are shown here. When $N_D = 1$, surprisal values are more sensitive to changes of $f_D$ because the information used to compute surprisal is then rather limited. Results (panels C and D in particular) show that surprisal values are rather robust: increasing or decreasing $f_D$ by the factor of two still yields correlation values above 0.9 for both Amazon and Delicious data.

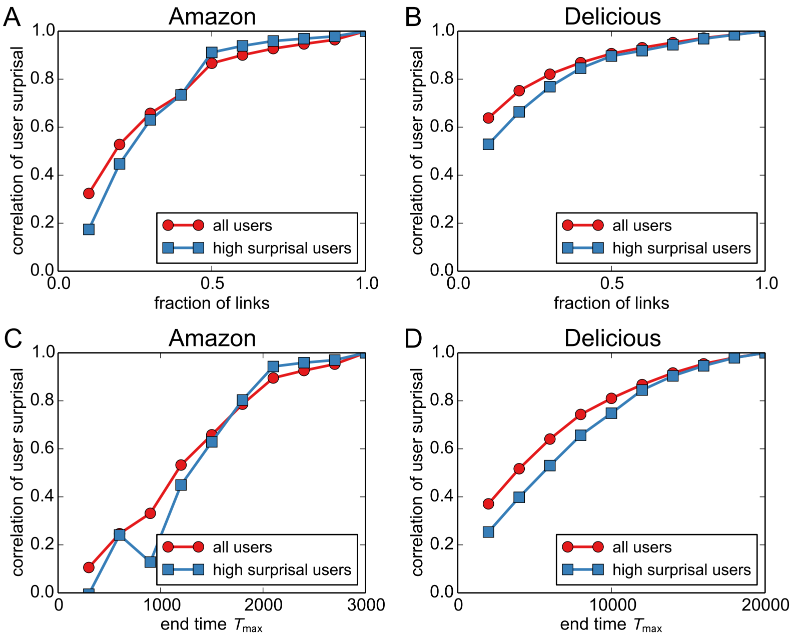


Figure S4: **Stability of user surprisal values with respect to the data.** We compute here the vector of user surprisal values $\boldsymbol{S}^*$ using the full data and measure its correlation with the vector of user surprisal values $\boldsymbol{S}$ obtained on data that ends after a certain fraction of links (panels A and B) and data that ends at a certain time (panels C and D). As in Fig. S3, the correlation value is computed for all users as well as for users whose surprisal value in $\boldsymbol{S}^*$ is greater than 10. Results in panels A and B show that even when one half of links is omitted (note that we omit here the most recent links, not the oldest), correlation between $\boldsymbol{S}^*$ and $\boldsymbol{S}$ is still around 0.9. Correlations decreases faster in panels A and B than in panels C and D because the speed at which new links are added increases with time in the studied systems. Setting the end time to $T_{\max}$ thus amounts to using less than $T_{\max}/T_W$ of all links ($T_W$ is the length of the whole dataset).

It is also possible to investigate the stability/persistence of user surprisal values by dividing the input data into four (for example) disjoint parts and comparing the surprisal values obtained in each of them. Since surprisal values are naturally noisy for low-degree users, we consider only users who have at least 10 links in respective data parts. When computing the Pearson correlation over all six different pairs of data parts, we obtain $0.67 \pm 0.08$ and $0.49 \pm 0.06$ for the Amazon and Movielens, respectively (the range given is the standard error of the mean). This confirms that there is substantial persistence in users' discovery patterns. Nevertheless, the correlation is not perfect—one can thus consider to study users' "success" periods and improve, for example, the degree prediction results by ignoring the users who are not sufficiently successfull discoverers lately.

# S1 Basic analytical results for the proposed network model

Denoting the fraction of fitness-driven users as $\mu_F := U_F/U$, we can write the following continuum equation which describes the evolution of the average degree of item $\alpha$ (see [1, 2] for more details on the continuum approximation approach)

$$\begin{aligned}\frac{\partial\langle k_\alpha(t)\rangle}{\partial t} = \mu_F\,\frac{f_\alpha\,A(t-\tau_\alpha)}{\sum_\beta f_\beta A(t-\tau_\beta)} + \\ + (1-\mu_F)\,\frac{(k_\alpha(t)+C)A(t-\tau_\alpha)}{\sum_\beta (k_\beta(t)+C)A(t-\tau_\beta)}\end{aligned} \tag{1}$$

where the two terms represent the contribution of the fitness- and popularity-driven users, respectively. The presence of the aging factor $A(\cdot)$ allows us to replace the sums in fraction denominators with their average values to which the sums approach at the time scale given by the form of $A(\cdot)$ and then fluctuate around them. In particular, we have

$$\begin{aligned}\sum_\beta (k_\beta(t)+C)A(t-\tau_\beta) \to \Omega_k,\\ \sum_\beta f_\beta A(t-\tau_\beta) \to \Omega_f.\end{aligned} \tag{2}$$

Equation (1) can now be solved analytically and yields the asymptotic result

$$\langle k_\alpha(\infty)\rangle = \left(\mu_F\,\frac{f_\alpha}{\Omega_f} + (1-\mu_F)\frac{C}{\Omega_k}\right)\frac{\mathrm{e}^{(1-\mu_F)T/\Omega_k}-1}{(1-\mu_F)/\Omega_k} \tag{3}$$

where $T = \int_0^\infty A(t)\,\mathrm{d}t$. Results for the previous model with preferential attachment, fitness and aging presented in [2, 3] are recovered by setting $\mu_F = 0$ and replacing $T$ with $Tf_\alpha$. We see that the expected final degree of items is indeed proportional to item fitness (the proportionality factor is given by the fraction of leaders in the system).

We finally note that one can devise a model with continuously-distributed user ability $a_i \in [0,1]$ where the two aforementioned item-choosing equations can be merged in one. We have studied the multiplicative form

$$P_{i\alpha} \sim f_\alpha^{a_i}\,(k_\alpha(t)+1)^{1-a_i}\,A(t-\tau_\alpha) \tag{4}$$

which implies that users of ability one respond only to item fitness, users of ability zero respond only to item popularity, and there is a continuous spectrum of user behavior between these two boundary ability values. However, we find the binary model with two discrete user groups easier to interpret and more amenable to analytical solution.

# References


[1] R. Albert, A.-L. Barabási, *Reviews of Modern Physics* **74**, 47 (2002).

[2] M. Medo, G. Cimini, S. Gualdi, *Physical Review Letters* **107**, 238701 (2011).

[3] D. Wang, C. Song, A.-L. Barabási, *Science* **342**, 127 (2013).
